# Activation thresholds, not quitting thresholds, account for the low prevalence effect in dynamic search

**DOI:** 10.3758/s13414-024-02919-1

**Published:** 2024-07-08

**Authors:** Mark W. Becker, Andrew Rodriguez, Jeffrey Bolkhovsky, Chad Peltier, Sylvia B Guillory

**Affiliations:** 1 Department of Psychology, Michigan State University, East Lansing, MI 48824 USA; 2 Naval Submarine Medical Research Laboratory (NSMRL), Groton, CT 06349 USA; 3 Leidos, Inc, New London, CT 06320 USA

**Keywords:** Continuous visual search, Dynamic visual search, Low-prevalence effect, Multiple targets

## Abstract

The low-prevalence effect (LPE) is the finding that target detection rates decline as targets become less frequent in a visual search task. A major source of this effect is thought to be that fewer targets result in lower quitting thresholds, i.e., observers respond target-absent after looking at fewer items compared to searches with a higher prevalence of targets. However, a lower quitting threshold does not directly account for an LPE in searches where observers continuously monitor a dynamic display for targets. In these tasks there are no discrete “trials” to which a quitting threshold could be applied. This study examines whether the LPE persists in this type of dynamic search context. Experiment 1 was a 2 (dynamic/static) x 2 (10%/40% prevalence targets) design. Although overall performance was worse in the dynamic task, both tasks showed a similar magnitude LPE. In Experiment 2, we replicated this effect using a task where subjects searched for either of two targets (Ts and Ls). One target appeared infrequently (10%) and the other moderately (40%). Given this method of manipulating prevalence rate, the quitting threshold explanation does not account for the LPE even for static displays. However, replicating Experiment 1, we found an LPE of similar magnitude for both search scenarios, and lower target detection rates with the dynamic displays, demonstrating the LPE is a potential concern for both static and dynamic searches. These findings suggest an activation threshold explanation of the LPE may better account for our observations than the traditional quitting threshold model.

## Introduction

Lab-based studies of visual cognition often utilize visual search tasks. One of the reasons that so many studies use this method is because it is a versatile task with many factors that can be manipulated to probe the allocation of attention (Wolfe & Horowitz, 2017). For instance, to investigate basic mechanisms of visual attention, researchers can manipulate aspects such as the set size (Palmer, 1995; Wolfe, 2010), the extent to which the search is an efficient feature based search or an inefficient conjunction search (Donner et al., 2002; Treisman & Gelade, 1980), or the specificity of the search templates (Malcolm & Henderson, 2009; Maxfield & Zelinsky, 2012). Researchers can also use visual search to investigate learning implicit or explicit learning processes. For instance, people implicitly learn to use repeated layouts to guide search (Chun, 2000; Chun & Jiang, 1998). Also, associating a non-task-relevant feature with reward can improve search speed (Anderson et al., 2012). Finally, changing the likelihood of features (Wolfe et al., 2003) or locations (Walthew & Gilchrist, 2006) associated with a target can influence search guidance. In short, visual search tasks have been instrumental to the development of some of the most prominent models of visual attention (Treisman & Gelade, 1980; Wolfe, 2021).

Visual search is intuitively appealing as a lab-based task because it resembles tasks from everyday life and thus seems to have ecological validity. For instance, on a given day, a person may search for their phone, keys, wallet, etc. Given the similarities between lab-based tasks and real-world searches, it would be beneficial if laboratory knowledge could be used to improve real-world searches. This is particularly relevant to the many real-world search scenarios that have important, life-saving consequences, i.e., searching radiological scans for cancer, baggage for weapons, or surveillance videos for potential threats. However, there are some differences between these real-world scenarios and typical lab-based search tasks, which can limit the ability to generalize from lab-based to real-world tasks (Clark et al., 2012).

In lab-based search tasks, targets are often frequent, typically appearing on 50% (in detection tasks) or 100% (in discrimination tasks) of trials. By contrast, targets are exceedingly rare in many important real-world search tasks, such as cancer screenings, baggage screenings, and submarine sonar detection. It has been estimated that only about .3% of scans show tumors in mammography screenings (Gur et al., 2004) and, while the true prevalence rate of weapons in airport luggage is unknown, airport security reports just one gun per 116,394 screenings (Fishel et al., 2015). It is suspected, though unknown, that enemy submarines appearing on friendly sonar monitors are also rare. The difference between high and low target prevalence rates can have significant impacts on search performance. Recent work investigating visual search with infrequent targets has found that target detection performance plummets as targets become rare – the low prevalence effect (LPE) (Ishibashi et al., 2012; Mitroff & Biggs, 2014; Peltier & Becker, 2017b; Van Wert et al., 2009; Wolfe et al., 2005). In the lab, researchers have found that a target that appears in 50% of trials is detected twice as often as when that same target only appears on 10% of trials (Peltier & Becker, 2016). Further, it appears that this effect grows as target prevalence decreases further, as prevalence and hit rate are logarithmically correlated (Mitroff & Biggs, 2014).

Investigations of the LPE have found that deficits in target detection come primarily from two sources: a decrease in quitting threshold and a conservative shift in decision-making criteria (Hout et al., 2015; Peltier & Becker, 2020; Wolfe & Van Wert, 2010). A major factor driving the LPE is the lowering of the trial-wide quitting threshold. When targets are rare, observers look at fewer items before making a target-absent response. This decreases the probability of seeing a target before responding target absent, and results in faster target-absent responses and increased misses (Hout et al., 2015; Peltier & Becker, 2016). The other factor is a conservative shift in the decision-making criterion used to determine whether a scrutinized object is a target or not. This process can be modeled as a shift in the criterion in signal detection theory (Green & Swets, 1974) or a shift in the decision thresholds of a drift diffusion account (Ratcliff & Rouder, 1998). In either case, the shift increases the amount of evidence required to identify an item as a target, thereby increasing the likelihood of misidentifying a target as a distractor. One eye-tracking study with rare targets (10% prevalence rate) found that search errors – i.e., misses attributable to never inspecting a target due to a low quitting threshold – accounted for 76% of misses, with the remainder due to identifications errors, i.e., misses attributable to inspecting a target but failing to recognize and report it due to a conservative decision-making criterion (Peltier & Becker, 2016).

Importantly, the evidence for the LPE has come almost exclusively from experiments in which the stimuli are static images. The image is presented at the beginning of a trial, and typically remains visible until the participant makes a present/absent decision, which typically terminates that trial and leads to a new trial with a new image. It is under these static scenarios that a significant portion of the LPE can be attributed to changes in the trial-level quitting threshold (Wolfe & Van Wert, 2010). As targets become rare, the quitting threshold is reduced and fewer items are inspected prior to a target-absent response, leading to increased misses and fast target-absent reaction times (RTs).

However, not all important real-world searches involve static individual scenes. For example, sonar operators, radar operators, and people analyzing video surveillance are tasked with searching for targets in continually changing dynamic displays. Continuous tasks are those in which viewers do not make target-absent responses to terminate a display, but continually monitor a stream of information and respond only when a target appears (Chan & Chan, 2022). In these dynamic searches, where there are no individual “trials” and no target-absent responses, there is no obvious role for a shift in quitting threshold to impact search performance. This raises the question of whether the LPE that is so robust with static images would generalize to a continuous, dynamic visual search scenario. Based on the eye-tracking data of Peltier and Becker (2016), if search errors from a decreased quitting threshold are no longer a factor, the LPE should be dramatically reduced. To test this prediction, we aimed to compare the magnitude of the LPE in a continuous task with dynamically moving stimuli to static search tasks with the same stimuli.

However, there are two relevant methods that may provide insight into whether we should expect to find a LPE in these types of continuous searches with dynamically moving stimuli. One involves research investigating search performance when stimuli are stationary but move dynamically through feature space, and the second is research investigating vigilance tasks. Muhl-Richardson et al. (2018a, 2018b) discussed real-world tasks, such as monitoring displays that color-code the density of geological formations, in which the color of a given location changes dynamically through color space. In these tasks a given location may drift through color space, eventually becoming the “target” color. While Muhl-Richadson et al.’s primary focus was to investigate whether the trajectory of the drift through feature space allowed predictive monitoring to guide attention (and eye movements) to locations likely to become the target, they did have one experiment that manipulated target prevalence. While they failed to find evidence that target prevalence influenced search accuracy, they noted that this failure to find a LPE may have resulted because the total time that objects were targets, relative to overall trial time, was low in both their moderate and low prevalence conditions (Muhl-Richardson et al., 2018b). Chan and Chan (2022) also had spatially fixed stimuli drift through color space during a continuous detection task. They were specifically interested in how target frequency impacted search performance. In contrast to Muhl-Richardson et al., they found evidence for a robust LPE despite the fact that these tasks did not allow target-absent responses to terminate a trial, thereby eliminating the effect that changes in a trial-wide quitting threshold might have on target detection performance. Given that Chan and Chan’s research was designed to investigate target prevalence effects and that Muhl-Richardson et al. suggested that their method may have obscured a potential LPE effect, the limited research on this topic suggests that dynamic search with no quitting thresholds may be susceptible to the LPE.

Both Muhl-Richardson et al. and Chan and Chan’s dynamic search tasks are similar to many vigilance tasks. The typical vigilance task requires participants to continuously monitor a display for a prolonged period of time while detecting sporadic targets, resulting in performance decreases over time, the vigilance decrement (for review, see Neigel et al., 2020). While our goal was not to investigate this vigilance decrement,^1^ vigilance experiments that have investigated target detection rates as a function of the target prevalence rate may inform our research on how target prevalence may impact search performance in continuous tasks. Colquhoun and Baddeley (Baddeley & Colquhoun, 1969; Colquhoun & Baddeley, 1964) varied target prevalence rates (called signal probability in that research domain) while observers monitored a series of arrays of circles and responded whenever one of the circles in an array was slightly larger than the others. Observers made target-present responses but not target-absent responses, with the search arrays automatically changing to a new array after a set period of time. As such, there were no quitting thresholds associated with making a target-absent response (although it is possible that observers quit their search once they have evaluated the display and simply wait for the next display to re-engage). Even so, they reported results akin to the LPE – the likelihood of missing a target increased as the number of arrays containing targets became rare. It is notable that they reported something like a LPE even in a task for which there was no ability to terminate individual trials, and thus no ability for a trial-wide quitting threshold to impact search accuracy.

Here we investigate the extent to which the LPE that has been well documented with static displays persists with dynamically moving search stimuli. Experiment 1 includes static and dynamic search conditions in which subjects search for a single target that appears at moderate or low prevalence rates. Experiment 2 extends this approach to a search for two targets: one that appears with moderate prevalence and the other that occurs at low prevalence. While we anticipate lower overall target detection performance with dynamically moving search stimuli (Kunar & Watson, 2011), to the extent that a major portion of the LPE depends on changes in trial-level quitting thresholds, we predict that the magnitude of the LPE effect should be reduced for dynamic stimuli. This is because the dynamic task eliminates the ability to quit an individual trial early, thereby eliminating the influence of a shift in quitting threshold. Instead, the only source of a higher miss rate under the low prevalence dynamic condition would be the change in decision making criterion where identification errors are more likely. This pattern would exhibit itself as an interaction between prevalence and search condition such that there is a greater difference in hit rate between high and low prevalence static searches than in dynamic searches. However, the findings of an LPE-like effect from the vigilance tasks and tasks with stimuli dynamically moving through feature space, which also eliminate the ability to quit individual trials, raises the possibility that we will find LPE effects despite having no trial-wide quitting thresholds in our dynamic task. If we continue to find a similar magnitude LPE in the dynamic task as the static task, it would require us to consider alternative explanations of the role that quitting thresholds play in the LPE.

## Experiment 1

### Methods

#### Participants

Previous literature investigating differences between low and moderate prevalence rates with static stimuli reported a very large effect size of d = 1.7 (Godwin, Menneer, Riggs, Cave, & Donnelly, 2015). The current study used dynamic displays and online data collection which might reduce the effect size; thus, we used a much more conservative effect size for our power calculations, using an f effect size of .325, halfway between the conventional cutoffs for moderate (.25) and larger effect sizes (.4). Power calculations for the main effects and interaction of an ANOVA with four groups, where power = .95, and an f-effect size of .325 yielded a required minimum total sample size of 126 (Faul et al., 2007). We then oversampled participants to allow for attrition due to online presentation of stimuli.

Participants were recruited online via the Prolific platform (https://www.prolific.co/). This platform allowed us to restrict recruitment to participants in the USA who were running the program on a desktop or laptop computer (rather than a tablet or phone). We recruited 242 total participants, of whom 235 completed the task (117 in the dynamic condition and 118 in the static). Given the online data collection method, we filtered our data in two ways. For the dynamic conditions, the program could drop frames of the display if the participants’ computer was not fast enough or had too many programs active in the background, as this reduced the browser’s ability to keep up with the dynamic displays. When this occurred, it could interfere with the correct tracking of conditions and result in the wrong number of trials per condition. An initial triage was performed to eliminate data from participants whose trial counts in any condition deviated by more than 5% from the expected number of trials, leading to the elimination of data from 25 participants. This issue of dropped trials did not occur for the static trials. This data filtering eliminated participants based on their hardware not presenting the dynamic stimuli accurately but given the online nature of data collection we also sought to identify participants that may not have been engaged with the task. To do so, we first investigated whether anyone pressed the same button throughout the entire task; two participants did so (both from the dynamic condition), and their data were eliminated from further analysis. We then used a recursive Mahalanobis distance method to identify participants whose multivariate data were outliers, based on a χ^2^ criterion of .001.^2^ This led to the elimination of data from 12 participants (ten in the dynamic and two in the static condition). Thus, the final sample on which analyses were preformed consisted of 193 participants, with 80 in the dynamic condition (39 in the low and 41 in the moderate prevalence condition) and 113 in the static condition (56 in the low and 57 in the moderate prevalence condition).

#### Design and displays

**Fig. 1:**
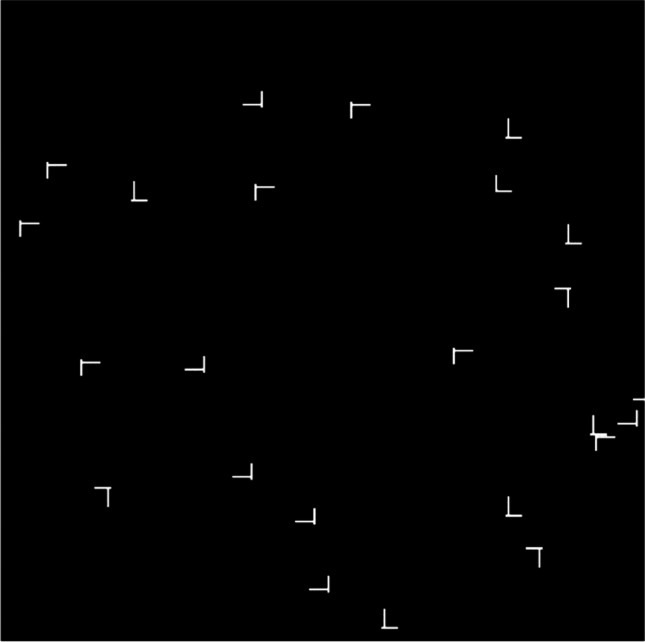
A screen grab from the dynamic displays of Experiment 1. In this example the “L” target appears in an upright orientation in the upper right quadrant. In the real experiment, each of the items would be moving along its own trajectory. In this example one element at the middle of the right side of the screen is exiting the screen, while the lowest item has just drifted onto the screen from the bottom

The experiment was a 2 (prevalence: high/low) x 2 (presentation type: static/dynamic) between-subjects design. The experiment was programmed in Psychopy and run on the Pavlovia (https://pavlovia.org/) platform online. Given this online presentation method, it is difficult to tightly control the size of the stimuli across participants. However, to mitigate this issue, the program began with a size scaling routine that asked people to adjust the height and width of an onscreen rectangle so that it matched the size of a credit card held up to the screen (Li et al., 2020 (Fig. 1a); Morys-Carter, 2021). The size of the participant’s adjustment was used to scale all stimuli in the experiment so that they were roughly equivalent across participants, although variability still occurred as we could not control the viewing distance of the participant. The visual sizes presented here are thus only estimates, based on the assumption that the participant accurately adjusted the rectangle during the scaling procedure and then viewed their screen from 57 cm. Screen refresh rates were set by the program to be 60 Hz.

Participants in the static condition performed 240 trials that were similar to traditional visual search trials. Each trial consisted of a display of 24 items. Participants were randomly assigned to search either for Ts or Ls and randomly assigned to the moderate prevalence or low-prevalence target conditions. In the low prevalence condition, 24 trials (10%) consisted of 23 distractors and one target. In the moderate target condition 96 trials (40%) consisted of 23 distractors and one target. The remaining trials (90% and 60%, respectively) were target-absent trials in which all 24 items were distractors. Distractors were “offset Ls,” in which the vertical line was placed halfway between the center of the horizontal line (where a T would intersect) and the end of the horizontal line (where the L would intersect). Each stimulus subtended ~ 1.2˚ x 1.2˚ of visual angle and could appear randomly in four possible orientations (rotated 0˚, 90˚, 180˚, or 270˚). The location of the 24 elements in the array were randomized with no constraints, which allowed items in the array to overlap. Although this overlap could occasionally make it difficult to identify a target, this was done to mimic realistic dynamic conditions. The static displays mimicked a single frame of the animation of the dynamic displays (see Fig. 1). Participants were asked to press the “M” key with their right index finger as soon as they detected a target, and to press the “Z” key if they determined that there was no target present. However, to mimic dynamic display conditions, the key presses did not terminate the display. Instead, displays remained on the screen for 6 s and then were replaced with the next array. This replacement occurred at 6 s regardless of whether the participants had made a button press or not.

In the dynamic task, participants pressed a button to indicate that a target was detected; there was no button to indicate target absent. Additionally, the stimuli were presented differently. The dynamic condition used one long animation comprised of 241 epochs of 6 s each. The epochs were used for data analysis purposes and were undetectable by subjects. During the first epoch, a new distractor entered the display aperture every 250 ms. It then drifted across the screen for 6 s before leaving the aperture. At the end of the first epoch the display had 24 items moving across the screen. Since their starting times were distributed, every 250 ms one element left the aperture as a new element entered simultaneously. Thus, after the first epoch the animation always displayed 24 visible items. When each item appeared, it followed one of 640 possible trajectories. These trajectories were defined by selecting a starting point from the 32 possible starting points, equally spaced along each edge of the square aperture. For a given starting point there were 24 possible ending points, i.e., the eight possible starting points on each of the other three sides of the aperture. However, four of these trajectories (the four nearest end points to the starting point) would have resulted in a trajectory with a length less than half the length of the square aperture, so these were eliminated from possible trajectories. Thus, for each of the 32 starting points there were 20 possible end points, creating 640 possible trajectories. Each element was randomly assigned to one of these trajectories and its speed was adjusted so that it would take ~6 s to complete the journey across the screen (the approximation due to rounding errors when dividing the distance of the trajectory by the 360 frames in 6 s).

After the initial epoch, this display consisted of 24 items moving smoothly across the screen for 24 minutes (240 epochs x 6 s each), with old items constantly exiting and new items constantly appearing. To mimic the static task across these 240 epochs, the low prevalence condition had 24 epochs with a target and the moderate condition had 96 epochs with a target. The target containing epochs were randomly distributed throughout the task. Again, the subjects in the dynamic condition were randomly assigned to either search for Ts or Ls.

There was no performance-based feedback for any conditions, but there was an auditory click to indicate any button press.

### Results

#### Accuracy

**Fig. 2:**
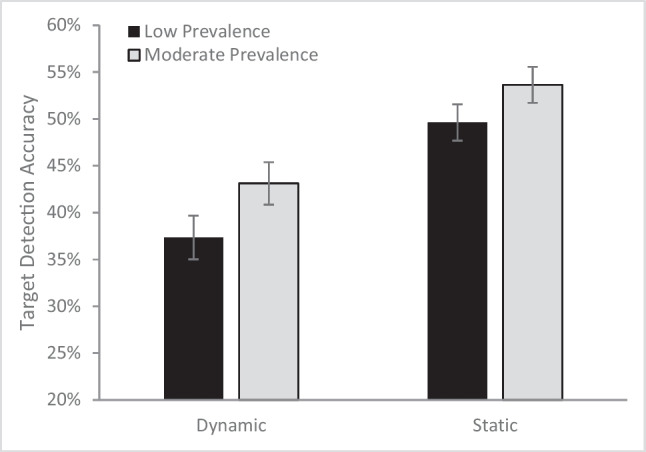
Target-present accuracy as a function of presentation type and prevalence rate. Error bars depict the standard error of the mean

An omnibus analysis of variance (ANOVA) with target-present accuracy as the dependent variable and prevalence rate (moderate or low) and presentation type (static or dynamic) as fixed effects (see Fig. 2) found a main effect of presentation type (*F*[1, 189] = 28.89, *p* <.001, µ_p_^2^= .13) with higher accuracy for the static (*M* = 51.6%, *SE* = 1.4%) than dynamic displays (*M* = 40.2%, *SE* = 1.6%). There was also a main effect of prevalence rate, (*F*[1, 189] = 5.32, *p* = .022, µ_p_^2^ = .027), with higher accuracy for the moderate prevalence targets (*M* = 48.4%, *SE* = 1.5%) than the low-prevalence targets (*M* = 43.5%, *SE* = 1.5%). The two factors did not interact (*F* [1, 189] = .17, *p* = .68, µ_p_^2^ = .001). The significant main effect of presentation type demonstrates that targets are more difficult to detect in the dynamic than the static search task, confirming past research (Kunar & Watson, 2011). In addition, the finding of a significant prevalence effect with no prevalence by presentation type interaction suggests that there is a similar LPE for both dynamic and static displays. The additive impact of prevalence rate and method of presentation, produced very low detections rates for low-prevalence targets presented in a dynamic display (*M* = 37.3%, *SE* = 2.3%), highlighting the potential difficulty in finding rare targets among dynamic displays, a concerning trend when considering real-world searches.

We performed a similar analysis on false alarm rates. There was a main effect of presentation type, (*F*[1, 189] = 80.31, *p*<.001, µ_p_^2^= .30), with higher false alarms in the static condition (*M* = 18.7%, *SE* = 1.2%) than the dynamic condition (*M* = 1.6%, *SE* = 1.5%).^3^ There was no detected main effect of prevalence rate nor a prevalence rate by presentation type interaction (both *F*[1, 189] < 1.9, both *p* > .16, both µ_p_^2^< .01).

### Reaction time (RT)

RT was defined as the time from the onset of the display to response in the static case, and from when the target first appeared on the screen until the button press in the dynamic condition.

**Fig. 3:**
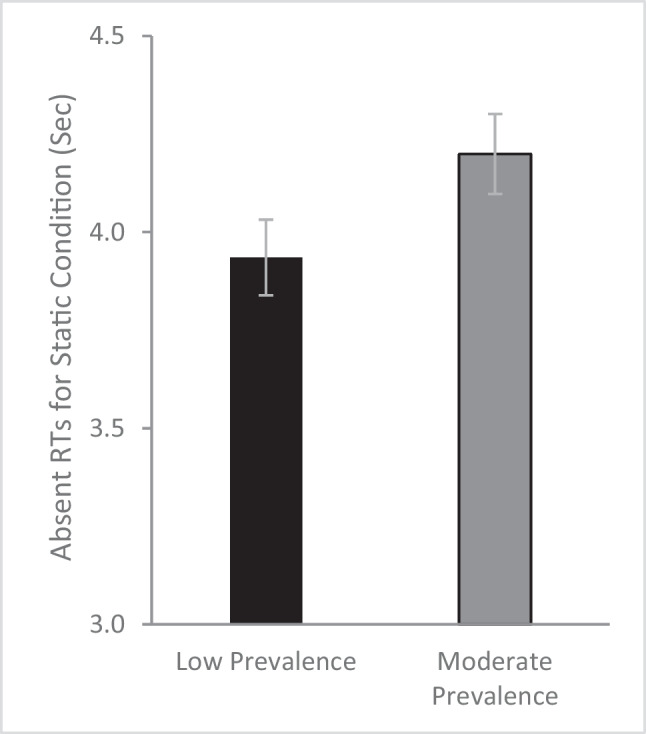
Target-absent reaction times (RTs) for the static conditions as a function of prevalence rate. Error bars depict the standard error of the mean

In search with static displays, a major factor responsible for the LPE is a decrease in the trial-level quitting threshold, indicated by faster target-absent reaction times for the low prevalence condition. Consistent with a shift in quitting thresholds, an independent t-test on target-absent RTs for the static search condition (Fig. 3), revealed significantly faster target-absent RTs (*t*[111] = 1.89, *p* = .031 one-tailed, *d* = .35) for the low prevalence (*M* = 3.94, *SE* = .10) than the moderate prevalence (*M* = 4.20, *SE* = .10) condition. Thus, even though the button press did not terminate the trial, the target-absent RTs suggest that low-prevalence targets result in lowered quitting thresholds for static displays. We note that there are no target-absent RTs for the dynamic condition since it is a continuous task where people only respond when they see a target. Furthermore, given the continuous nature of the dynamic condition, it does not make sense to try to evaluate a trial level quitting threshold.

**Fig. 4:**
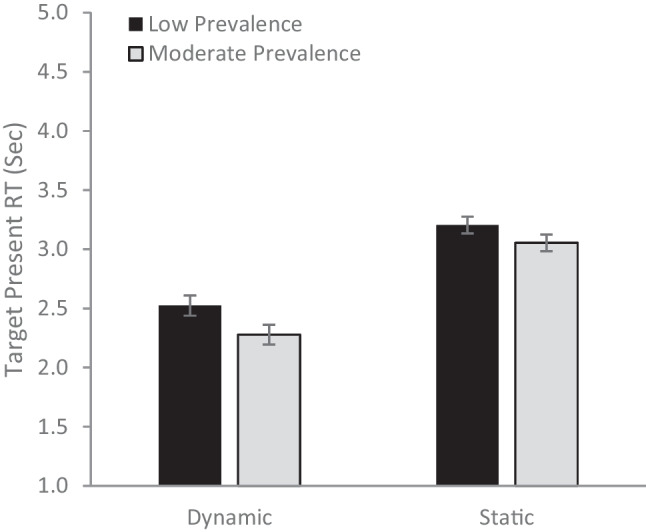
Target-present reaction time (RT) as a function of presentation type and prevalence rate. Error bars represent the standard error of the mean

Finally, we ran an analysis of target-present RTs, including the presentation type and prevalence rate as fixed effects (Fig. 4). There was a main effect of prevalence rate (*F*[1, 189] = 6.55, *p* =.011, µ_p_^2^= .033), with faster RTs for the moderate (*M* = 2.67, *SE* = .05) than the low (*M* = 2.86, *SE* = .06) prevalence condition. There was a main effect of presentation type (*F*[1, 189] = 88.88, *p* <.001, µ_p_^2^= .32), with much faster RTs for the dynamic (*M* = 2.41, *SE* = .06) than the static (*M* = 3.24, *SE* = .06) displays. The two factors did not interact (*F*[1, 189] = .38, *p* = .54, µ_p_^2^= .02).

### Discussion

In Experiment 1, static display conditions replicated the standard LPE and we found that misses increased and target-absent RTs decreased when targets became rare (Ishibashi et al., 2012; Peltier & Becker, 2016). This pattern of results has traditionally been used to argue that the LPE results from rare targets producing lowered quitting thresholds, which then results in a less thorough search of the display before responding target absent. This leads to an increase in miss rates and faster target-absent RTs.

We also found evidence for an LPE in dynamic search. Miss rates were higher for the low prevalence than the high-prevalence target conditions. Also, the lack of a significant target prevalence rate by presentation type interaction suggests that the magnitude of the LPE is similar for static and dynamic displays. The presence of an LPE in the dynamic search task is difficult to explain based on a shift in quitting thresholds – the dynamic task had no distinct trials to which a quitting threshold could be applied.

Finally, although the magnitude of the LPE was similar for both presentation types, overall target detections were reduced for dynamic displays relative to static displays. This finding of low overall detection rates with dynamic search is consistent with prior work that had discrete search trials comprised of mixed static and moving stimuli, which found lower detection rates when the target was one of the moving stimuli (Kunar & Watson, 2011). However, in Kunar and Watson (2011) they also found slower target detection RTs for moving stimuli, but in this study, we found faster target-present RTs in the dynamic than static presentation conditions. This difference could result from the fact that all stimuli were moving in our dynamic condition, but only half of the stimuli were moving in their experiments (and half were static). This may have biased people to spatially select static items more than moving items. However, the difference in false alarm rates for the two presentation methods complicates the interpretation of the lower overall target detection rates for the dynamic condition, something we consider more fully in the general discussion.

In our experiments we can imagine two possible explanations for fast target detection RTs in the dynamic search condition. The first is that there may be a bias to attend to novel or new items (Johnston et al., 1990). This would mean that targets in the dynamic condition would be found early in their trajectories or not at all. A second possibility is that participants could not track all 24 moving items (Pylyshyn, 2001; Pylyshyn & Storm, 1988), and rather than trying to shift their gaze between each item in the display, they may have adopted a strategy of keeping their gaze somewhat central in the display, evaluating items as they approached the middle. Given that many items drift near the center of the screen, this “staying in place” approach could be a reasonable strategy. Using this strategy, RTs might shift faster because the participants are not making many eye-movements, which can be inefficient (Smilek et al., 2006; Zelinsky & Sheinberg, 1997).

**Fig. 5:**
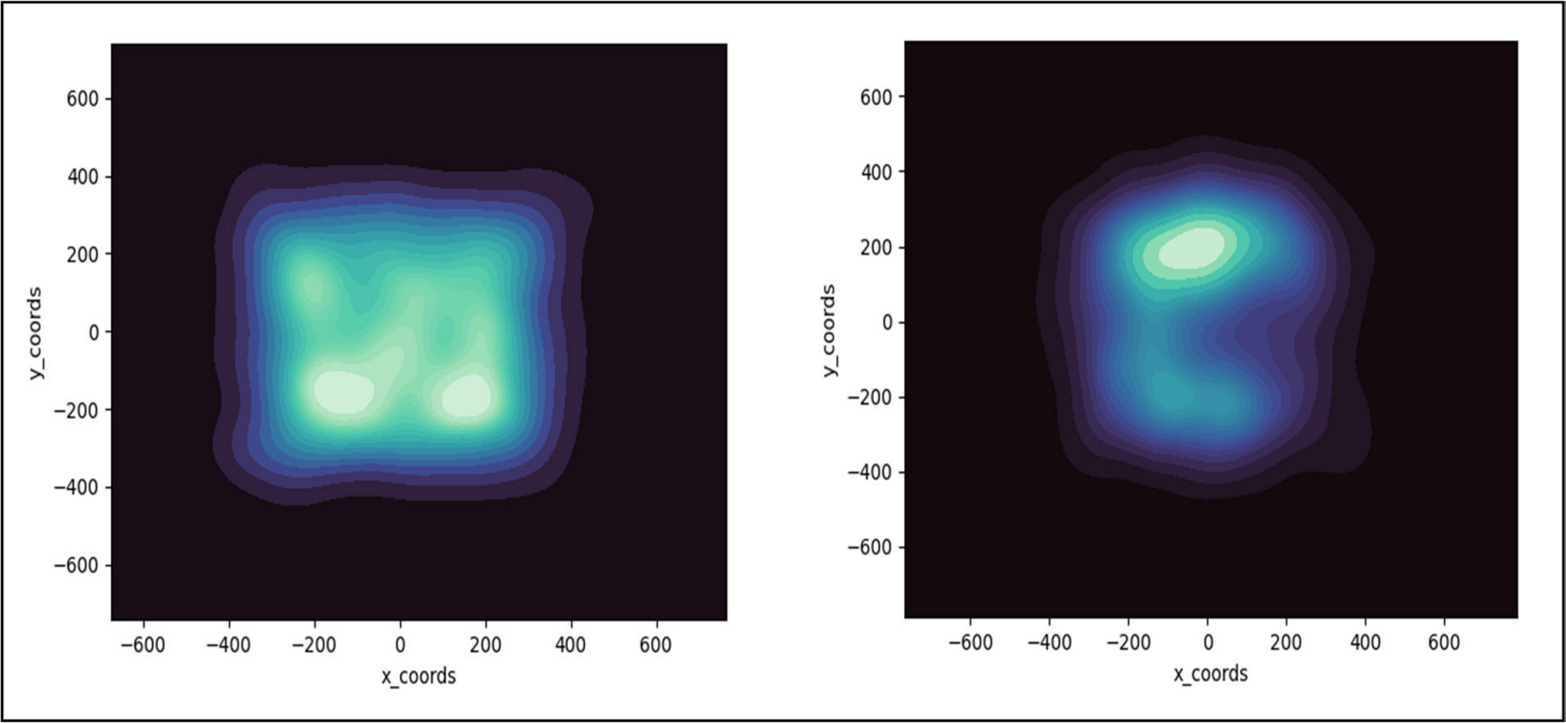
Heatmaps of the location of detected targets in static displays (**left panel**) and dynamic displays (**right panel**) of Experiment 1

These two alternatives make differential predictions about the spatial distribution of detected targets (hits) in the dynamic condition. The novelty hypothesis suggests that target detections in the dynamic condition should occur near the edges of the display, with relatively few hits in the central portion of the display. The staying in place hypothesis makes the opposite prediction – target detections should be concentrated in the center of the display with few targets detected near the edge. To evaluate this issue, we generated heat maps of the detected targets’ locations. For dynamic displays, we determined the location occupied by the moving target when the target-present button press occurred. For comparison, we also generated heat maps of the locations of targets that were successfully detected in the static displays (Fig. 5).

For the static search conditions, targets are found relatively uniformly across a large square region of the display, although there is a clear central bias consistent with past research (Buswell, 1935; Mannan et al., 1996). The pattern is quite different for the dynamic search condition – detected targets were highly concentrated in the upper central part of the display, with a slight bias toward the left. This dynamic pattern is inconsistent with the novelty hypothesis and is instead consistent with the staying in place hypothesis. While eye-tracking would be required to definitively evaluate whether observers adopt a stay in place strategy for the dynamic displays, the clear difference between the static and dynamic heat maps suggested that subjects are engaging in different processes across the two types of searches. In the static case subjects are more actively searching throughout the display for targets, while in dynamic displays they may be adopting a more passive method of evaluating items when they reach a particular location. Given these striking differences and the fact that the dynamic condition is inconsistent with a quitting threshold explanation, it is surprising that both methods produce LPEs of similar magnitudes.

Before speculating on why the LPEs might be similar across these different types of search environments, we wanted to replicate and extend this finding. To that end, in Experiment 2 we used a different method of manipulating prevalence rate (Hout et al., 2015). Participants searched for both Ts and Ls and the two targets appeared at different prevalence rates. This allowed us to maintain a relatively high overall prevalence rate of 50%, but with one target that was rare (10%) and a second that was moderately prevalent (40%). Despite the overall high prevalence rate, these types of manipulations have shown LPEs for the rare target (Godwin et al., 2010, 2015; Menneer et al., 2012). Again, we implemented this prevalence manipulation in both a static and dynamic search scenario to determine whether the LPE would be similar across search scenarios.

## Experiment 2

### Method

#### Participants

Participants were again recruited online via the Prolific platform (https://www.prolific.co/). The platform allowed us to restrict recruitment to participants in the U.S. who were running the program on a desktop or laptop computer (rather than a tablet or phone). In total, 147 participants completed the experiment, with 82 in the static version and 65 in the dynamic task. Again, some participants’ computers had difficulty keeping up with the dynamic task, resulting in the incorrect number of trials per condition. As in Experiment 1, we eliminated the data from participants who had trial counts in a condition that deviated by more than 5% from the expected number, leading to the elimination of data from 21 subjects. The static condition did not tax participants’ computers and all participants received the correct number of trials. Like Experiment 1, we also eliminated the data from participants based on the same recursive Mahalanobis distance, leading to the elimination of data from one participant in the dynamic task and nine participants in the static task. The analyses were performed on the data from the remaining 73 subjects in the static condition and 42 subjects in the dynamic condition. While this rate in the static task is high, it might reflect that participants did not like having to wait for the next trial and were attempting to advance the display by responding (despite the button press not terminating the trials).

### Design and displays

The experiment was a 2 (prevalence: high/low) x 2 (presentation type: static/dynamic) mixed design with prevalence as a within-subjects factor and presentation type as a between-subjects factor. The static and dynamic displays were identical to Experiment 1 with the exception that in each condition, there were 120 target-present trials (or epochs for the continuous task), with 96 trials displaying one target and 24 displaying the other target. The target that was displayed more frequently was randomly assigned by participants. The remaining 120 trials were target-absent trials. As in Experiment 1, the distractor was an offset L, such that the junction between the two perpendicular lines was halfway between a target T and a target L, thus equally similar to both targets. There were never two targets on the display at the same time during either condition. Given the two possible targets, the method of responding also changed. Participants were asked to press one key to indicate the presence of a “T” and a second key to indicate an “L.” To make the static conditions as similar to dynamic conditions as possible, the method of responding was identical, and participants were informed to withhold responding if no target was present. Like with the dynamic case, key responses in the static case did not terminate the trial, and participants were informed that the display would automatically switch to a new display after 6 s. In this way the static closely matched the dynamic displays in terms of the amount of time that the target was visible and in terms of the method of responding.

### Results

Our main dependent variables were the percentage of correct target detections, false alarm errors, and the RT needed to correctly identify a target. In the static condition, RT was defined as the time from the onset of the display to response, and from when the target first appeared on the screen until the button press in the dynamic condition. For both dependent variables we performed a 2 (presentation type: static/dynamic) x 2 (target prevalence: moderate/low) mixed model ANOVA with presentation type as a between-subjects variable and target prevalence as a within-subjects variable.

#### Accuracy

**Fig. 6:**
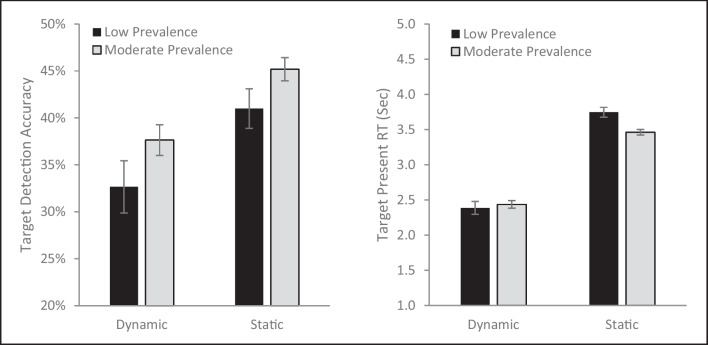
The mean hit accuracy (**left panel**) and reaction time (RT) for hits (**right panel**) for Experiment 2 are plotted as a function of prevalence rate and presentation type. Error bars are the standard error of the mean

For accuracy (Fig. 6, left panel) there was a main effect of target prevalence (*F*[1, 113] = 5.13, *p* <.025, µ_p_^2^= .043), with better target detection for the moderate prevalence target (*M* = 41.4%, *SE* = 1.0%) than the low-prevalence target (*M* = 36.8%, *SE* = 1.7%). There was also a main effect of presentation type (*F*[1, 113] = 15.41, *p* <.002 , µ_p_^2^= .12), with better target detections for static (*M* = 43.1%, *SE* = 1.2%) than dynamic displays (*M* = 35.1%, *SE* = 1.6%). The two factors did not interact (*F*[1, 113] = .38, *p* = .85, µ_p_^2^ <.001). Again, the prevalence rate and presentation type appeared to be additive, leading to particularly poor target detection (*M* = 32.7%, *SE* = 2.8%) with a low-prevalence dynamic target. Like Experiment 1, false alarms were higher in the static (*M* = 24.6%, *SE* = 2.8%) than the dynamic (*M* = 8.5%, *SE* = .04%) method of presentation (*F*[1, 113] = 12.13, p < .001, µ_p_^2^= .097), complicating the interpretation of the overall lower hit rate in dynamic conditions.

#### RT

For RT (Fig. 6, right panel) there was a main effect of presentation type (*F*[1, 113] = 273.71, *p* <.001, µ_p_^2^= .71), with faster RTs for the dynamic (*M* = 2.41 s, *SE*=.06) than static condition (*M* = 3.60 s, *SE* = .04). The main effect of prevalence rate was marginally significant (*F*[1, 113] = 3.61, *p* = .06, µ_p_^2^= .031). However, these results were qualified by a presentation type by prevalence interaction (*F*[1, 113] = 7.32, *p* = .008, µ_p_^2^ = .061). Follow-up paired t-tests on the simple effects reveal that this interaction occurred because there was not a significant difference as a function of prevalence rate for dynamic displays (t[41] = .60, *p* = .55, *d* = .09), but numerically the more prevalent target (*M* = 2.44 s, *SE* = .06) was found more slowly than the less prevalent target (*M* = 2.39 s, *SE* = .08). By contrast, for static displays moderate prevalence targets (*M* = 3.46 s, *SE* = .03) were detected significantly (t[72] = 3.53, *p* < . 001, *d* = .41) more quickly than low-prevalence targets (*M* = 3.75 s, *SE* = .07).

### Discussion

In this experiment the overall prevalence rate was fairly high (50%), but subjects searched for two targets, one that appeared at moderate (40%) and one that appeared at low (10%) prevalence rates. With this type of prevalence manipulation, a simple explanation of the LPE based on changes in a trial-level quitting threshold would be difficult to apply – even in the static case (Hout et al., 2015) – because there is no change in overall prevalence, thus there should be no change in quitting threshold or search errors. Even so, the results from Experiment 2 generally replicate those from Experiment 1.

The Experiment 2 accuracy data clearly show that the LPE occurs for both static and dynamic search stimuli. In both presentation types, the lower prevalence targets yielded significantly more misses than the targets that appeared more frequently. Furthermore, the fact that target prevalence rate did not interact with presentation type suggests that the magnitude of the low prevalence effect is roughly the same for static and dynamic search tasks. We can therefore conclude that the LPE is a concern for tasks in both static and dynamic searches when the overall prevalence of targets is somewhat high, but specific targets appear infrequently.

As in Experiment 1, we found lower overall detection accuracy for dynamic compared to static search displays. This dynamic cost seemed to be additive with the LPE, which suggests that dynamic search for low-prevalence targets is particularly likely to result in missed targets, i.e., target detection rates as low as 30% in our task.

**Fig. 7:**
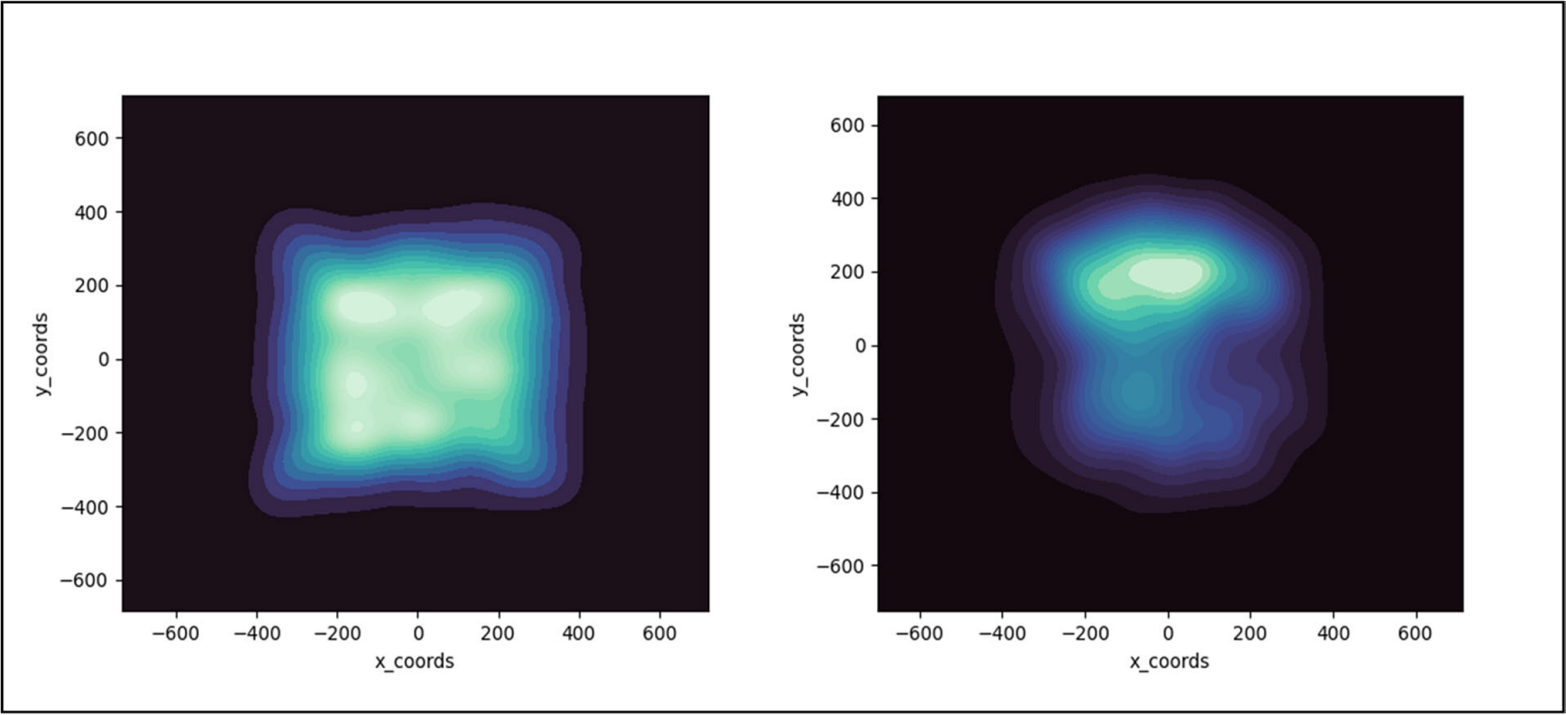
Heatmaps of the location of detected targets in static displays (**left panel**) and dynamic displays (**right panel**) of Experiment 2

Also like Experiment 1, we found RTs for hits were faster in the dynamic versus static displays. To determine whether this was due to novelty bias or a stay-in-one-place strategy, we again made heat maps of the locations of the detected targets (Fig. 7). These heat maps are very similar to those in Experiment 1 and again suggest dramatically different search processes for the static and dynamic searches. The static map suggests a more complete search of a large region of the display and the dynamic map suggests a stay-in-one-place approach to monitoring the display.

We found strikingly similar LPEs for static and dynamic searches, and the results of Experiment 2 were quite similar to those of Experiment 1, despite implementing prevalence rate in a different way.

## General discussion

We found strikingly similar LPEs for both static and dynamic search tasks across two experiments that established target prevalence rates differently. The fact that these effects were so similar across experiments and search tasks highlights that the LPE is extremely robust and occurs in a variety of contexts. Further, we believe that the similar magnitude of the LPEs across these experiments and conditions suggest that a common mechanism may be responsible for the decrease in target detections for rare targets. However, the question remains about what that mechanism may be. One of the main mechanisms posited to be responsible for the LPE is a change in quitting thresholds. As targets become rare in a static search, quitting thresholds are reduced and people search the displays less thoroughly before responding target absent, thereby producing higher miss rates and faster target-absent reaction times (Peltier & Becker, 2016, 2017a; Shi et al., 2020; Wolfe & Van Wert, 2010). However, quitting thresholds cannot adequately explain our results, as there is no opportunity to quit a trial with a target-absent response in our continuous dynamic searches. Even so, our results are generally consistent with results from vigilance tasks (Baddeley & Colquhoun, 1969; Colquhoun & Baddeley, 1964) and tasks with stationary stimuli that move drift through feature space (Chan & Chan, 2022), which found evidence for a LPE even though the methods did not afford an opportunity to quit individual trials. It is also worth noting that other experiments (Hout et al., 2015; Peltier & Becker, 2016) have also found the LPE in rapid serial visual presentation of stimuli, where there is also no termination of a trial, and thus a simple quitting threshold explanation of the LPE may be inadequate.

We can envision two explanations for the LPE that may not depend on a shift in the trial-wide quitting threshold. The first is that there may be a shift in the item-by-item decision criteria used to evaluate whether each item is a target or distractor. Using static displays, we previously argued (Peltier & Becker, 2016) that low-prevalence targets produced a starting-point bias toward the distractor boundary in a drift diffusion model of this item-by-item decision process. That work included eye-tracking and found that low-prevalence search conditions resulted in briefer dwell times on distractors and longer dwell times on targets, providing evidence that there was a bias toward the distractor boundary in the drift-diffusion model. A similar explanation was offered by Wolfe et al., (2022), who suggested an adaptive timer for the item-by-item inspection process. Under that explanation, items may be missed because there is inadequate time allocated to the decision process and thus a target may fail to reach its threshold for awareness. Assuming low prevalence results in a faster timer, few targets will reach that threshold and more items will be missed.

Both these explanations based on shifts to the item-by-item inspection process could account for the LPE and do not rely on a trial-level quitting threshold. As such, both may account for the present findings of sizeable LPE in our continuous dynamic search task that does not provide for a trial-level quitting threshold. However, we are reluctant to suggest that these shifts in the item-by-item inspection process can account for the entirety of the LPE we observed. Our reticence follows from our earlier work (Peltier & Becker, 2016) showing that, with static displays, low-prevalence rates also decrease the number of items inspected prior to making a target-absent response and that a vast majority (~75%) of misses in that work were attributable to search errors (errors where the target was never fixated) rather than inspection errors (errors where the target was fixated and still missed). Both of these findings suggest that the LPE is at least partially driven by a shift in how many items are selected for scrutiny – how many items make it into the item-by-item decision process.

In the classic two stage model of the LPE (Chun & Wolfe, 1996; Wolfe & Van Wert, 2010), this selection process is driven by an activation threshold that gates which items are selected for further scrutiny by the item-by-item decision mechanism. According to the model by Chun and Wolfe (1996), there is parallel analysis across the visual scene that results in an activation map in which an individual item’s activation level is determined through a combination of its bottom-up saliency and its similarity to the features of a search template. That is, very salient items and items that share features with the to-be-detected target will have high activation levels in the initial activation map. An activation threshold is then applied to the activation map so that only items with activation levels higher than the activation threshold are selected for evaluation during a second, more serial phase where each item is attended and evaluated via the item-by-item decision mechanism. If during that process an item is evaluated to be the target, the trial is terminated with a target-present response. But if all items above the activation threshold are evaluated with none being identified as a target, a target-absent response is executed.

This model can account for the LPE by having the target prevalence rate alter the activation threshold; if targets are rare the activation threshold increases such that fewer items are fewer items are scrutinized before making a target-absent response, increasing the likelihood of a miss and resulting in faster target-absent RTs. That is, the increase in the activation threshold leads to a reduction in the trial-level quitting threshold, leading to the LPE.

However, the driving mechanism behind the trial-level quitting threshold is the activation threshold that impacts attentional selection. In static search this activation threshold dictates which locations and elements are scrutinized. But, in dynamic displays, the constant movement of the items may make it difficult to engage in that type of selection of potential target locations. Instead, based on our heatmaps, it seems participants adopt a “stay in place” strategy and evaluate items once they drift into the zone that is being monitored. If we assume that this region is relatively large, given the complexity of our displays, there are likely multiple items simultaneously within that region, thus there may be a need to prioritize some items over others for attentional selection. We posit that the same activation threshold theorized to gate selection of spatial locations in static displays is applied to items in the attended region during dynamic displays. If so, the activation threshold would dictate which items within the central attended region are selected for attentional scrutiny and which are not. If low-prevalence targets produce an increase in this activation threshold, fewer items will exceed the threshold in dynamic search, thereby resulting in an increased likelihood of missing a target. In short, we believe that the increase in the activation threshold responsible for the LPE in static search may also be responsible for the LPE in dynamic search. The only difference is that in static search, the threshold determines which items/locations are scrutinized in whole image search, while in our dynamic search task the threshold dictates which items will be selected for scrutiny once they drift into the monitored region.

In short, in line with earlier two-factor models of the LPE (Wolfe & Van Wert, 2010), we posit that the LPE in both static and dynamic search are impacted by two factors, and both of those factors are similar in dynamic and static searches. Low target prevalence rates likely result in changes to the item-by-item decision mechanism. These changes could be thought of as a bias toward the distractor boundary in a drift diffusion model, or as a reduction in the time allotted to the item-by-item decision process. Either process would produce a reduction in the number of targets successfully identified. However, based on our earlier work we believe these changes to the item-by-item decision mechanism likely does not fully account for the LPE, and a substantial portion of the LPE is attributable to changes in a selection mechanism that gates which objects are evaluated by the item-by-item decision mechanism. We posit that the activation threshold is responsible for this selection process. In static displays the activation threshold dictates which locations and how many locations are scrutinized, with only those locations having items with activations above the threshold being attended. In our dynamic displays, spatial selection may be more difficult, and instead we posit that attention is allocated to a relatively fixed region of space and items that drift within that region are selected only if their activation exceeds the activation threshold. Thus, the same activation threshold guides selection in both scenarios, thereby producing similar magnitudes of LPE.

Even though the LPE was similar for dynamic and static tasks, in both experiments we found that target detection rates were overall lower in the dynamic relative to the static search displays. While this finding is consistent with prior evidence showing lower detection rates for moving relative to static stimuli (Kunar & Watson, 2011), in our dynamic conditions the lower target detection rates were accompanied by much lower false alarm rates. Thus, this finding likely results from a criterion shift rather than a shift in sensitivity, such that much more evidence was required to identify an item as a target in the dynamic method than in the static. Indeed, if one were to analyze corrected hit rate (hits-false alarms) rather than analyzing hits and false alarms separately, for both experiments the direction of the significant main effect for presentation method would be reversed (Exp. 1: *F*[1, 189] = 6.58, *p* =.01, µ_p_^2^= .03; Exp. 2: *F*[1, 113] = 3.93, *p* = .05, µ_p_^2^=.03], with higher accuracy in the dynamic (Exp. 1:* M* = 38.7%, *SE* = 1.8%; Exp. 2: 26.6%, *SE* = 3.3%) than static conditions (Exp. 1: *M* = 33.2%, *SE* = 1.5%; Exp. 2: *M* = 18.5%, *SE* = 2.5%). In short, it appears that participants were very conservative in the dynamic displays, having a high threshold/criterion for identifying a target. This high threshold produced a lower number of hits and far fewer false alarms relative to static displays with the same stimuli. From a practical perspective, whether this criterion shift with dynamic displays is desirable or not depends on the cost of misses and false alarms in a particular task. If misses are catastrophic, this conservative shift is problematic as the proportion of misses increases. However, if false alarms are more problematic than misses, this type of criterion shift would be welcomed.

It is worth commenting on the location of the detected target locations for the dynamic condition. First, the same upper, slightly left bias occurred for both experiments. Second, these maps were generated collapsing across subjects, and to find this type of consistency across subjects suggests a relatively universal bias; if different subjects chose random different regions to monitor, once one collapsed across subjects there would be a relatively uniform heatmap throughout the display. While we were somewhat surprised to find this relatively universal bias, there are existing data that have reported this attentional bias. Specifically, Feng and Spence (2014) found an upper visual field advantage for detecting targets among distractors using an attentional visual field task. Upper visual field advantages have also been noted in visual search (Previc & Blume, 1993), although that study also found a slight right-side advantage versus the left side advantage we found. Other researchers have suggested that the ventral attentional network, thought to be responsible for orienting attention toward salient and unexpected events, is right hemisphere dominant, producing a bias for information in the left visual hemi-field (Corbetta et al., 2008; Corbetta & Shulman, 2002). Thus, this finding of a relatively consistent bias toward the upper left visual field in dynamic search is not without precedence. From a practical standpoint, it suggests that targets are more likely to be missed in dynamic displays if they remain in the lower visual field.

Finally, there are some limitations to this work. Foremost is the lack of monitoring of our research subjects, as they participated in the task remotely, introducing concerns about viewing distance, distraction, and equipment, among others. Additionally, without eye-tracking our subjects, we cannot rule out the possibility that observers “quit” dynamic search by looking offscreen or ceasing to search at times, or that differences in performance between conditions are attributable to an as yet unknown pattern of search or identification errors in dynamic search. Last, the work here was a first step to comparing and exploring the effects of prevalence on dynamic and static search, as well as multiple target dynamic and static search. Future research should seek to replicate and extend this line of research with additional prevalence rates, real-world stimuli, and stimuli that move in random patterns rather than only straight lines.

## Conclusions

Our main objective was to determine whether the LPE, a well-documented and robust finding from static visual search tasks, would occur in a continuous dynamic search task. Given that the predominant theory for the LPE in static visual search is that a low prevalence rate reduces quitting thresholds, resulting in less thorough searches before responding target-absent, it was uncertain whether the effect would occur in a dynamic task that did not have discrete trials that subjects quit. That said, across the two experiments that implemented low prevalence in different ways, we found that magnitude of the LPE was similar across dynamic and static visual search tasks. We posit that although the two search conditions produce very different search strategies, they both rely on attentional selection of the target. That attentional selection process is dependent on whether the target’s activation in an early visual representation is above the activation threshold. If low prevalence rates raise this activation threshold higher, in both types of search fewer targets will exceed the threshold, resulting in a similar LPE in both tasks. Thus, the LPE is a concern for performance in both dynamic and static search tasks. In addition, relative to static search, dynamic search performance was reduced for both moderate and low-prevalence targets. The combination of these two effects lead to extremely poor target detections in dynamic visual search, highlighting the potential dangers of performing dynamic visual search for low-prevalence targets. From an applied perspective, this additive effect, where low prevalence dynamic searches yield the lowest target detection rates, is concerning. Real-world critical tasks, such as security and sonar monitoring, may be vulnerable to both effects, raising concerns about how such tasks are constructed, how the observers are trained, and how performance is monitored. While there have been many experimental manipulations that have attempted to reduce the LPE in static search, few have succeeded. Given that the combined effect of prevalence and motion is a new research topic, despite its critical applications, interventions to improve search under these conditions are necessary and urgent. Researchers should investigate the applicability of the interventions that have successfully reduced the LPE in static searches for dynamic low prevalence searches, and potentially develop novel methods.

## Data Availability

The data generated and analyzed during the current study may contain proprietary information or restricted access information. Data may be available upon written request to Naval Medical Research Command (NMRC) Legal subject to approval.

## Footnotes

1 It is worth noting that a meta-analysis by See et al. (1995) investigated the magnitude of vigilance decrements as a function of the types of experimental tasks used. Based on their classification scheme, the type of task we performed here (what they would classify as a simultaneous, low target rate, cognitive task) should not produce a vigilance decrement (mean effect size for these types of tasks of .00).

2 To compute the Mahalanobis distance, we used each subject’s mean for each of the calculated dependent variables. For the static condition, this included the false alarm rate, hit rate, target-absent RT, and hit RTs. For the dynamic condition, there was no target-absent RT, so we only used the three remaining dependent variables.

3 False alarm rates were quite high in the static condition of both experiments. We believe that these high false alarm rates may have resulted because occasionally two distractors overlapped in such a way to create an image that appeared like a target. These problematic overlaps could also occur in the dynamic condition, but in that condition the overlap was fleeting.
